# Prevalence, Awareness, Treatment, and Control of Hypertension among Chinese First-Generation Migrants and Italians in Prato, Italy: The CHIP Study

**DOI:** 10.1155/2017/6402085

**Published:** 2017-04-13

**Authors:** Pietro A. Modesti, Maria Calabrese, Ilaria Marzotti, Hushao Bing, Danilo Malandrino, Maria Boddi, Sergio Castellani, Dong Zhao

**Affiliations:** ^1^Department of Experimental and Clinical Medicine, University of Florence, Florence, Italy; ^2^Diabetology Unit, Ospedale Misericordia e Dolce, Prato, Italy; ^3^Associazione Culturale Cinese di Fujian in Italia, Prato, Italy; ^4^Department of Epidemiology, Capital Medical University Beijing Anzhen Hospital and National Institute of Heart, Lung & Blood Disease, Beijing, China

## Abstract

Data on health needs of Chinese living in the South of Europe are lacking. To compare prevalence, awareness, treatment, control, and risk factors for hypertension between Chinese migrants and Italian adults, a sample of 1200 first-generation Chinese migrants and 291 native Italians aged 35–59 years living in Prato (Italy) was recruited in a community-based participatory cross-sectional survey. Primary outcome measure was hypertension, diagnosed for blood pressure values ≥ 140/90 mmHg or current use of antihypertensive medications. Associations with exposures (including age, gender, body mass index, waist, education level, total cholesterol, and triglycerides) were examined using logistic regression. When compared with Italians, Chinese had higher hypertension prevalence (27.2% versus 21.3%, *p* < 0.01), with comparable levels of awareness (57.4% and 48.4%) but lower treatment rates (70.6% and 90.0%, resp.). In both ethnic groups age and parental history of hypertension were predictors of awareness and treatment, body mass index being predictor of hypertension diagnosis. In Chinese participants, where the optimum cut-off point for body mass index was ≥23.9 kg/m^2^, the sensibility and specificity prediction for hypertension were 61.7% and 59.8%, respectively (area under the ROC curve = 0.629). Implementation of specific, culturally adapted health programs for the Chinese community is now needed.

## 1. Introduction

The distribution of cardiovascular risk factors, as well as the benefits of advances in prevention and treatment of chronic disease, are not shared equally across economic and ethnic groups either in the United States [1] or in Europe [2–4]. Cultural factors seem to play a primary role in limiting the efficacy of prevention strategies conceived for the native population in reaching first-generation migrants. This issue is now relevant for most EU countries where immigration flows importantly grew in recent decades [2]. As recently stated by the European Society of Cardiology [5], information on health needs of minority groups living in Europe is now available for subjects originating from Sub-Saharan African countries and South Asia [6, 7], whereas data for Chinese are still limited [8]. In recent decades China experienced a rapid increase in stroke incidence [9] and the control of hypertension and other risk factors is now recognized as a public health priority [10–12].

In the last decades migration flows from China to Europe have been mainly directed towards Italy and Spain [13], and Chinese are now the third largest overseas-born population in Italy [14]. In particular, Prato has the highest proportion of Chinese immigrants of any Italian province, subjects being mainly occupied in the textiles industry of the area [15]. Risk factor distribution in the Chinese community of Prato was recently investigated in the CHIP (CHinese In Prato) survey which enrolled Chinese first-generation migrants aged 18 to 59 years [16, 17]. However, data on the differences in hypertension burden between Chinese and Europid adults are lacking. This information is essential for health policies, strategies, and plans [18].

The present study was thus performed (1) to compare the prevalence of hypertension and other main risk factors between first-generation Chinese immigrants and Italian adults in the age group 35 to 59 years; (2) to investigate the relationship of hypertension with obesity indices; and (3) to identify the optimal BMI cut-off value to be adopted for screening purposes.

## 2. Methods

### 2.1. Setting, Study Design, and Participants

Located in Tuscany, 30 Km far from Florence, Prato has a population of more than 180,000 with a number of Chinese regular residents in the area constantly growing from 169 in 1990 to 15,957 in 2014 [19]. In 2014 the CHIP survey, incorporating principles of community-based participatory research [20, 21], was performed. More precisely a community-academic partnership, composed of the Consulate General of Florence, the four local community-based Chinese organizations, and the Chinese and Italian Universities, was built to develop a sensitive, culturally appropriate, no coercive recruitment, and enrolment process [19]. A network sampling procedure was adopted [16, 17, 22]. To be eligible for the present analysis, participants recruited in 2014 had (1) to self-identify to be born in continental China and to have grandparents born in that country; (2) to be between 35 and 59 years of age; and (3) to live permanently in Prato. In 2014 a cohort composed of native Italian population was randomly sampled from General Practice lists stratified by age and gender using an extraction program. Each subject was initially sent a letter informing them about the study, followed by an invitation to attend for screening. Subjects were replaced after two invitations. Those with whom no contact was established after three invitations were sent a letter by recorded delivery mail. Response rate of the eligible Italian subjects approached during recruitment was 67%. Exclusion criteria included pregnant women, critically ill individuals, and impaired cognitive ability as judged by clinical staff members.

### 2.2. Ethics, Consent, and Permissions

The study was approved by the Ethical Committee of the Azienda Ospedaliero-Universitaria Careggi (Ref. OSS.14.089). Written informed consent was obtained from all participants. Subjects were provided with a written description of the study in their choice of Chinese or Italian and written consent was obtained at time of entry from each participant. Participants with untreated clinical diseases identified during the examinations were advised to see their general practitioner or referred to the Hospital of Prato. No other incentives were offered to study participants. Data collected were anonymous and deidentified. The screening phase was performed between June 2014 and April 2015.

### 2.3. Data Collection

All participants were instructed to fast overnight before the day of survey. In the early morning (between 07.00 and 10.00 am) individuals attended the Research Centre where trained Chinese and Italian staff members administered a questionnaire and performed physical (blood pressure and anthropometry) and biochemical blood measurements (glucose, total cholesterol, and triglycerides).

Questionnaire gathered information on participant sociodemographic data, tobacco use, alcohol consumption, medical and reproductive history, medication use, and migration [17].

Blood pressure (BP) was measured three times using a clinically validated semiautomatic digital sphygmomanometer (M6; Omron Matsusaka Co. Ltd., Japan) with appropriate cuff size according to current guidelines [23]. The average of the last two readings was used for analysis. Body weight, height, and waist and hip circumferences were measured according to standardized protocols [24]. Waist-to-hip ratio was calculated as waist circumference (cm) divided by hip circumference (cm). Waist-to-height ratio was calculated as waist circumference (cm) divided by height (cm). Biochemical measurements were performed on finger-prick blood samples using validated dry chemistry methods (AccuChek AVIVA, Roche Diagnostics S.p.A., Mannheim, Germany for glucose and MultiCare-in, HPS, Italy, for total cholesterol and triglycerides) [25, 26]. Nonfasting participants were asked to return at fast for blood tests. Participants with fasting glucose ≥ 126 mg/dL were also asked to return for confirmatory testing. All requested participants attended the second visit.

### 2.4. Diagnostic Criteria

The primary outcome variable was the prevalence of hypertension, defined as systolic BP ≥ 140 mmHg, or diastolic BP ≥ 90 mmHg, or being on antihypertensive medication [23]. Awareness of hypertension was defined as self-report of any previous diagnosis of hypertension by a healthcare professional among participants with hypertension. Treatment of hypertension was defined as self-reported use of a prescription medication for management of hypertension at the time of survey. Control of hypertension was defined as antihypertensive treatment associated with average systolic and diastolic BP values < 140 mmHg and < 90 mmHg, respectively. Blood pressure was stratified according to the recommendations of the 2013 ESH-ESC guidelines (grades ESH-ESC) [23].

Diagnosis of diabetes mellitus (DM) was based on fasting plasma glucose criteria (≥126 mg/dL confirmed by repeat testing) and/or current treatment with glucose-lowering drugs [27]. High cholesterol was classified for total cholesterol levels ≥ 240 mg/dL [28, 29] and high triglycerides for triglycerides levels ≥ 200 mg/dL [29].

Other exposures included education level (no studies, primary and secondary school, high school, college, or more), alcohol use, smoking (current smokers and noncurrent smokers defined as those who never smoked and former smokers who quit smoking), health insurance (none, registration to National Health System, or private), Italian speaking (yes, no), BMI, and waist. For occupational classification of workers (excluding retired and unemployed), “blue collars” (workers who perform manual labors) and “white collar” (workers who perform professional job duties in an office setting) were considered.

### 2.5. Statistical Analysis

The sample size for comparison of Italian and Chinese groups aged 35–59 years was based on an estimated hypertension prevalence of 20% in Italian and 29% in Chinese populations. Considering a 5% confidence level (alpha error) and 80% statistical power (beta error), the estimated sample size was at least 281 individuals. Values are expressed as mean ± standard deviation (SD) or *n* cases (%). Analyses were stratified by 5-year age groups (35–39 years; 40–44 years; 45–49 years; 50–54 years; 55–59 years). Age standardized rates were based on direct standardization using the WHO World Standard Population [30]. Associations of hypertension with exposures were explored with logistic regression analysis. Exposures included age, sex, education categories, current smoking, high total cholesterol, high triglycerides, BMI, and waist-hip ratio tertiles (defined separately for each sex and ethnic group). When appropriate, test of hypothesis was done at significance level 0.05 two-sided. For regression analysis ORs and 95% CI were calculated. Receiver operating characteristics (ROC) curves for hypertension or undiagnosed hypertension and the areas under the curve were then calculated for BMI with corresponding 95% confidence intervals. The largest sensitivity-specificity product value obtained from each ROC curve was calculated. IBM SPSS software (version 22.0, SPSS Inc., Chicago, Illinois, USA) was used for analysis.

## 3. Results

### 3.1. Characteristics of Participants

Overall, 1200 Chinese and 291 Italian participants were investigated for the present study. Chinese participants had left China at an average age of 34.6 ± 8.2 years. Only 17.0% had lived in China urban areas, the large majority (83.0%) coming from rural China. In the Chinese cohort 201 subjects did not complete primary education, this condition being more prevalent among women than men (OR 2.35; 95% CI 1.69 to 3.27). Participants able to speak Italian were 29% with no differences by gender. Only 23% of investigated subjects had access to health services on a par with Italian citizens (subscription to the Regional Health System) with no differences by gender. Other 19% of participants self-reported the attribution of the Temporary Present Foreigner code with free temporary access (1 year) to healthcare. However, the large majority (58%) had no free access to the healthcare (private or public). Chinese participants were mainly occupied in light manual works in the textile industry (*n* = 1119, 93%), only a minority being housekeepers (*n* = 29; 2.4%), or manager or self-employed professionals (*n* = 18; 1.5%). Conversely, in the Italian cohort, only 60 participants (21%) were manual workers, 126 were manager or self-employed professionals (43.3%), and 87 (29.9%) were white-collar office worker. Participants unemployed and seeking work were 2 (0.2%) and 6 (2.1%) in the Chinese and Italian cohort, respectively. Overall, 450 out of the 635 women (71%) in the Chinese cohort had at least one previous abort for unwanted pregnancy versus 27 out of the 149 women (18%) of the Italian cohort (chi-square test, *p* < 0.001).

Main measurements in Italian and Chinese study participants are reported in Table 1. Chinese were significantly shorter and lighter and had lower BMIs than the Italian participants. Waist and hip circumferences and waist-to-hip ratio were also significantly smaller in Chinese. However, mean levels of diastolic BP, fasting glucose, total cholesterol, and triglycerides were significantly higher in Chinese than in Italian participants (Table 1).

### 3.2. Hypertension Burden in the Chinese and Italian Cohorts

**Figure 1:**
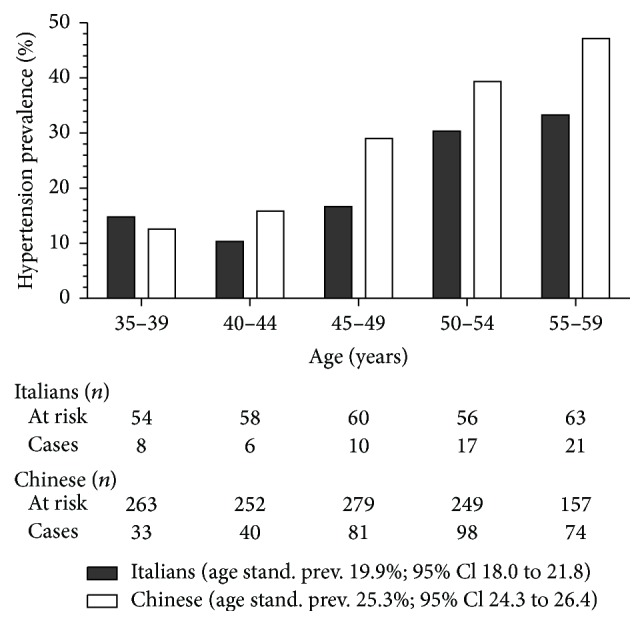
Age specific and age standardized (to WHO population 2001) prevalence of hypertension in the CHIP study population aged 35 to 59 years.

Overall, hypertension was diagnosed in 326 and 62 subjects among Chinese and Italian participants (27.2% and 21.3%, resp.). Age specific hypertension prevalence is reported in Figure 1. Age standardized prevalence was 25.3% (95% Cl 24.3 to 26.4) and 19.9% (95% Cl 18.0 to 21.8) in the Chinese and Italian cohorts, respectively. In the Italian cohort all the 62 participants with hypertension had Grade 1 HT whereas, in the Chinese cohort, 66 (24%) and 20 participants (7%) had Grade 2 and Grade 3 HT, respectively. Participants with hypertension aware of their condition were 187 (57.4%) in the Chinese and 30 (48.4%) in the Italian cohort (age- and sex-adjusted OR 1.53; 95% Cl 0.88 to 2.66). Although Chinese with hypertension aware of their condition were less frequently treated with drugs than native Italians (*n* = 132; 70.6% and *n* = 27; 90.0%, resp.; age- and sex-adjusted OR 0.23; 95% Cl 0.06 to 0.85), the rate of BP control did not differ between the two groups (*n* = 56, 42% and *n* = 12, 44% in the Chinese and Italian Cohort, resp.; age- and sex-adjusted OR 0.91; 95% Cl 0.39 to 2.13). Among subjects with hypertension belonging to the Chinese cohort, the use of antihypertensive drugs was independent from the registration to the Regional Healthcare system.

**Figure 2:**
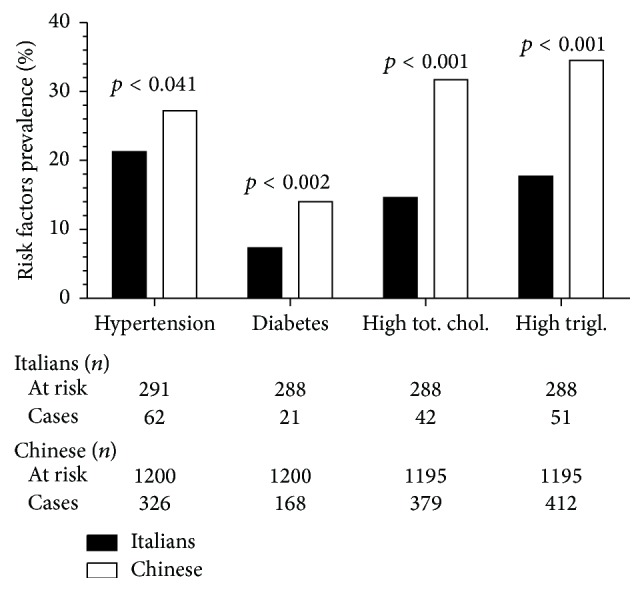
Prevalence of hypertension, diabetes, hypercholesterolemia (total cholesterol ≥ 240 mg/dL), and hypertriglyceridemia (triglycerides ≥ 200 mg/dL) in the Chinese and Italian cohorts.

Prevalence of all main risk factors was higher in the Chinese than in the Italian cohort (Figure 2) (Table 2). The OR for hypertension (Chinese versus Italians) further increased when education and work categories were included in the model (Model 2). When controlling also for obesity indices and other exposures a further increase was observed (Model 3) (Table 2).

At adjusted multivariable logistic regression only body mass index was associated with hypertension in both ethnic groups (Table 3). The overall ability of body mass index to correctly identify Chinese subjects with both hypertension and undiagnosed hypertension in the Chinese cohort was finally assessed with ROC curves. In the whole Chinese cohort, where the optimum cut-off points for body mass index were ≥23.9 kg/m^2^, the sensibility and specificity prediction for hypertension were 61.7% and 59.8%, respectively (Table 4). The areas under the ROC curves of body mass index for hypertension prediction were 0.629 and 0.597 in the whole Chinese cohort and among Chinese unaware of hypertension, respectively.

## 4. Discussion

According to the present findings, first-generation Chinese immigrants have a higher prevalence of hypertension and main risk factors than the Italian population independently from socioeconomic conditions. Around 2.8 million Chinese citizens currently reside legally in Council of Europe member States, with the largest groups being in Italy, France, Russia, and the United Kingdom. In the last two decades the presence of Chinese remained almost stable in Northern Europe whereas it importantly increased in Italy, mostly in the textile industry area near Florence. The limited studies comparing health needs of Chinese and Europid populations highlight the strength of the present study. In the Health Survey for England [31] the Chinese community living in UK had a prevalence of hypertension which was comparable to values we found in Chinese living in Italy. The characteristics of the two communities may differ, because the Chinese community now living in Prato is mainly composed of subjects born in China whereas most Chinese participants investigated in UK were born in Europe. On the other hand hypertension in the Europid cohort was more prevalent in UK than in Italy, so that, differently from what observed in UK [31], Chinese in Prato had higher prevalence of hypertension than native Italian population. These findings might let us hypothesize that Chinese migrants do not alter their original habits to follow the habits of the host country. In the same line it is to be considered that, among subjects with hypertension belonging to the Chinese cohort, the use of antihypertensive drugs was independent from the registration to the Regional Healthcare system or the capability to speak Italian. In particular rate of treatment was lower in the Chinese than in the Italian cohort, despite comparable levels of hypertension awareness. Specific prevention strategies have thus to be implemented in the Chinese community. Investing in a multiethnic perspective is thus necessary for eliminating inequities in risk factor control because prevention programs addressed to resident population might be inefficient for ethnic minorities [18, 32].

It has been consistently reported that risk factor distribution and health needs among different populations are markedly influenced by socioeconomic conditions [1]. The socioeconomic differences of the two investigated populations might influence observed results. In the present study Chinese participants were manual workers with low education level, mostly coming from rural China. The Italian sample is mainly composed of middle-class citizens. The two samples can be considered representatives of the two ethnic groups living in Prato. However, when socioeconomic indicators (education level and work) were included in the model the OR of Chinese for hypertension further increased. It is therefore conceivable that additional factors might play a role. Specific investigations on nutritional habits and sodium consumption are needed.

Overweight and obesity are established risk factors for hypertension [33, 34]. In the present survey BMI rather than central obesity was independently associated with hypertension in both Chinese and Italian cohort. Most importantly, notwithstanding the significantly higher prevalence of hypertension and other risk factors in Chinese than in the Italian cohort, Chinese participants were found to have markedly lower mean BMIs than the Italians. It is noteworthy that in Chinese participants the higher BMI was associated with hypertension with a cut-off value of 24 kg/m^2^. The WHO consultation group indeed recommended a lower cut-off of BMI for Asian with respect to native European populations [35] and the identified diagnostic cut-off for overweight in Chinese was 24 kg/m^2^ [36]. This information is important in the light of prevention because the different cut-off value for obesity have to be adopted by physicians who are facing the new patients [37]. The existence of a distinct cut-off value is also to be communicated within the Chinese community because the emulation of native population might lead migrants to increased risk.

## 5. Study Limitations

This study has several potential limitations.

First, the study design was cross-sectional, so we cannot conclude the cause-effect relationship.

Second, we are aware that the inclusion of undocumented migrants in the present surveys bears limitations. This group is usually excluded from epidemiological studies and surveys because the ability to go back to a list of individuals in some form is lacking. This is the reason why the sampling method of the study subjects differed between Chinese immigrants and Italian inhabitants. On this basis, it would be inappropriate to compare the two groups. However, in consideration of the limited availability of data regarding Chinese migration to the South of Europe, of the high presence of this ethnic group in the area, and of the fact that most EU countries currently offer emergency care to undocumented migrants, current insight on specific health needs of Chinese population in Italy might be important to offer health authority the opportunity to launch specific health programs [18]. The direct participation of the whole Chinese Community in the present shared project following the principles of a participatory research is to be acknowledged representing a proof of their willingness to collaborate in future actions of screening within the community.

Third, the sample size of Italians was limited to fully investigate age- and sex-stratified associations. In addition present survey investigated subjects in work ages of 35 to 59 years. We are aware that additional studies with larger sample sizes are needed to evaluate ethnic differences in hypertension burden, particularly at older ages. However, the creation of a cohort composed by young and middle aged subjects may offer the opportunity to follow these subjects in the future and to investigate the transition to the process of care in the Chinese community.

## 6. Conclusions

This study shows a high prevalence of hypertension and other main risk factors among young and middle-aged first-generation Chinese migrants settled in Italy. Present findings provide health authority useful information to respond to health needs of Chinese community and develop upstream specific preventive strategies.

## Figures and Tables

**Table 1:** Demographic data and selected clinical and laboratory findings in Italian and Chinese participants.

Variables	Chinese *n* = 1200	Italians *n* = 291	*p* value	Difference (95% Cl)
Age (years)	46.2 ± 7.1	47.6 ± 7.5	0.004	−1.4 (−2.3 to −0.4)
Height (cm)	163.1 ± 7.9	171.3 ± 10.7	0.001	−8.1 (−9.2 to −7.0)
Weight (kg)	63.3 ± 10.7	74.8 ± 12.2	0.001	−11.5 (−12.9 to −10.1)
Body mass index (kg/m^2^)	23.7 ± 3.1	25.4 ± 3.2	0.001	−1.7 (−2.2 to −1.3)
Hip circumference (cm)	95.4 ± 6.4	98.3 ± 12.2	0.001	−2.8 (−3.9 to −1.8)
Waist circumferences (cm)	82.7 ± 9.5	88.3 ± 13.3	0.001	−5.6 (−6.9 to −4.2)
Waist to hip ratio	0.866 ± 0.070	0.898 ± 0.077	0.001	−0.032 (−0.042 to −0.023)
Systolic BP (mmHg)	120.5 ± 19.2	120.4 ± 13.9	0.889	0.2 (−2.2 to 2.5)
Diastolic BP (mmHg)	80.3 ± 11.7	77.8 ± 10.9	0.001	2.5 (1.0 to 4.0)
Heart rate (bpm)	71.7 ± 10.2	70.9 ± 8.8	0.235	0.8 (−0.5 to 2.0)
Fasting glucose (mg/dL)	118.3 ± 33.4	103.6 ± 12.9	0.001	14.7 (10.8 to 18.7)
Total cholesterol (mg/dL)	233.1 ± 62.0	190.3 ± 55.5	0.001	42.8 (35.0 to 50.6)
Triglycerides (mg/dL)	194.9 ± 105.9	163.1 ± 86.8	0.001	31.8 (18.6 to 44.9)

Values are mean ± SD.

**Table 2:** Odds ratios (95% CI) for different factors (Chinese versus Italians).

	Model 1 OR (95% Cl)	Model 2 OR (95% Cl)	Model 3 OR (95% Cl)
Smoke (past or current)	0.62 (0.43 to 0.89)	0.64 (0.38 to 1.10)	0.74 (0.41 to 1.35)
Alcohol (yes)	0.37 (0.28 to 0.50)	0.45 (0.28 to 0.71)	0.38 (0.22 to 0.66)
Body mass index	0.86 (0.82 to 0.89)	0.85 (0.79 to 0.91)	0.93 (0.90 to 0.97)
Waist	0.95 (0.93 to 0.96)	0.94 (0.92 to 0.96)	1.03 (0.91 to 1.17)
Total cholesterol ≥ 240 mg/dL	3.07 (2.13 to 4.42)	2.36 (1.32 to 4.20)	1.65 (0.87 to 3.13)
Triglycerides ≥ 200 mg/dL	2.84 (2.01 to 4.00)	2.97 (1.70 to 5.19)	2.20 (1.18 to 4.09)
Hypertension	1.61 (1.16 to 2.24)	2.65 (1.47 to 4.76)	3.44 (1.71 to 6.94)

Model 1 adjusted for age and sex.

Model 2 adjusted for age, sex, education, and work.

Model 3 adjusted for age, sex, education, work, and all exposures included in the table.

**Table 3:** Association between exposures and hypertension at multivariate logistic regression (adjusted for all exposures reported in the table) performed among Chinese and Italian participants.

Ethnicity	Exposures	All subjects		Not aware of hypertension	
		OR (95% Cl)	*p* <	OR (95% Cl)	*p* <
Chinese	Age (years)	1.09 (1.07 to 1.12)	0.001	1.06 (1.03 to 1.10)	0.001
	Gender (male)	1.00 (0.73 to 1.37)	0.988	1.04 (0.68 to 1.58)	0.873
	Education level	1.01 (0.83 to 1.23)	0.960	1.01 (0.78 to 1.32)	0.935
	Work (white collar)	1.94 (0.95 to 3.97)	0.070	3.00 (1.34 to 6.75)	0.008
	Body mass index (kg/m^2^)	1.11 (1.04 to 1.19)	0.002	1.09 (1.00 to 1.20)	0.050
	Waist (cm)	1.02 (1.00 to 1.05)	0.091	1.01 (0.98 to 1.05)	0.460

Italians	Age (years)	1.03 (0.99 to 1.08)	0.184	1.00 (0.93 to 1.06)	0.865
	Gender (male)	1.23 (0.63 to 2.40)	0.546	1.53 (0.63 to 3.73)	0.348
	Education level	0.75 (0.41 to 1.38)	0.358	0.83 (0.36 to 1.90)	0.657
	Work (white collar)	1.10 (0.49 to 2.47)	0.817	0.63 (0.24 to 1.69)	0.363
	Body mass index (kg/m^2^)	1.26 (1.13 to 1.41)	0.001	1.28 (1.11 to 1.47)	0.001
	Waist (cm)	0.99 (0.96 to 1.01)	0.323	0.97 (0.94 to 1.00)	0.056

**Table 4:** Areas (with 95% confidence intervals) under the receiver operating curves and cut-off values for body mass index, for identifying hypertension in the whole Chinese cohort and in Chinese participants unaware of hypertension.

Sex	All Chinese participant				Chinese participants unaware of hypertension			
	Area	SE	(Area 95% CI)	Cut-off	Area	SE	(Area 95% CI)	Cut-off
Men	0.621	0.027	(0.568 to 0.673)	24.0	0.570	0.039	(0.494 to 0.646)	23.8
Women	0.634	0.024	(0.586 to 0.681)	23.9	0.619	0.033	(0.554 to 0.685)	23.6
All	0.629	0.018	(0.593 to 0.664)	23.9	0.597	0.025	(0.548 to 0.647)	23.7
